# Passive neck brace for surgeons

**DOI:** 10.1111/nyas.15308

**Published:** 2025-03-10

**Authors:** Zixiao Yang, Tejas S. Sathe, Meghal Shah, Jay Hemant Shah, David L. Hu

**Affiliations:** ^1^ Woodruff School of Mechanical Engineering Georgia Institute of Technology Atlanta Georgia USA; ^2^ Department of Surgery Columbia University Medical Center New York New York USA; ^3^ Medicine Department of Radiology and Department of Pediatrics Emory University Atlanta Georgia USA; ^4^ School of Biology Georgia Institute of Technology Atlanta Georgia USA

**Keywords:** brace, nuchal ligament, posture, surgical ergonomics

## Abstract

A surgeon peers downward into a body cavity when operating. Holding this position for hours across weeks, months, and years may lead to neck pain and musculoskeletal disorders. We were inspired by ungulates such as giraffes and horses, which use dorsal‐ventral flexion to graze for 9–14 h per day without perceivable neck pain. Ungulates evolved a strong nuchal ligament that relieves neck muscles by stretching to support some of the weight of the head during grazing or running. In contrast, humans evolved an upright posture, and like many primates, have a reduced nuchal ligament. The goal of this study is to use the nuchal ligament as inspiration for a neck brace that passively supports the weight of the head while still permitting lateral flexion, ventral‐dorsal flexion, and rotation. We assembled a prototype using an elastic band, headband, and back posture corrector. Our device augments the human nuchal ligament by using a stiff material and greater mechanical advantage. By our calculations, flexing the head ventrally 40 degrees when wearing the brace reduces the torque applied by neck muscles by 21%. Our device is a proof‐of‐concept that a bioinspired device can offload neck muscular tension and prevent injury.

## Graphical abstract

Surgeons peer downward into body cavities when operating. Holding this position for hours across weeks, months, and years may lead to neck pain and musculoskeletal disorder. Inspired by the strong nuchal ligaments of ungulates such as giraffes and horses, we analyzed the structure and strain of the nuchal ligament and proposed a neck brace that would augment the human nuchal ligament to offload neck muscular tension and prevent injury.

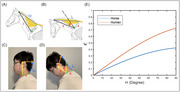

## INTRODUCTION

**FIGURE 1:**
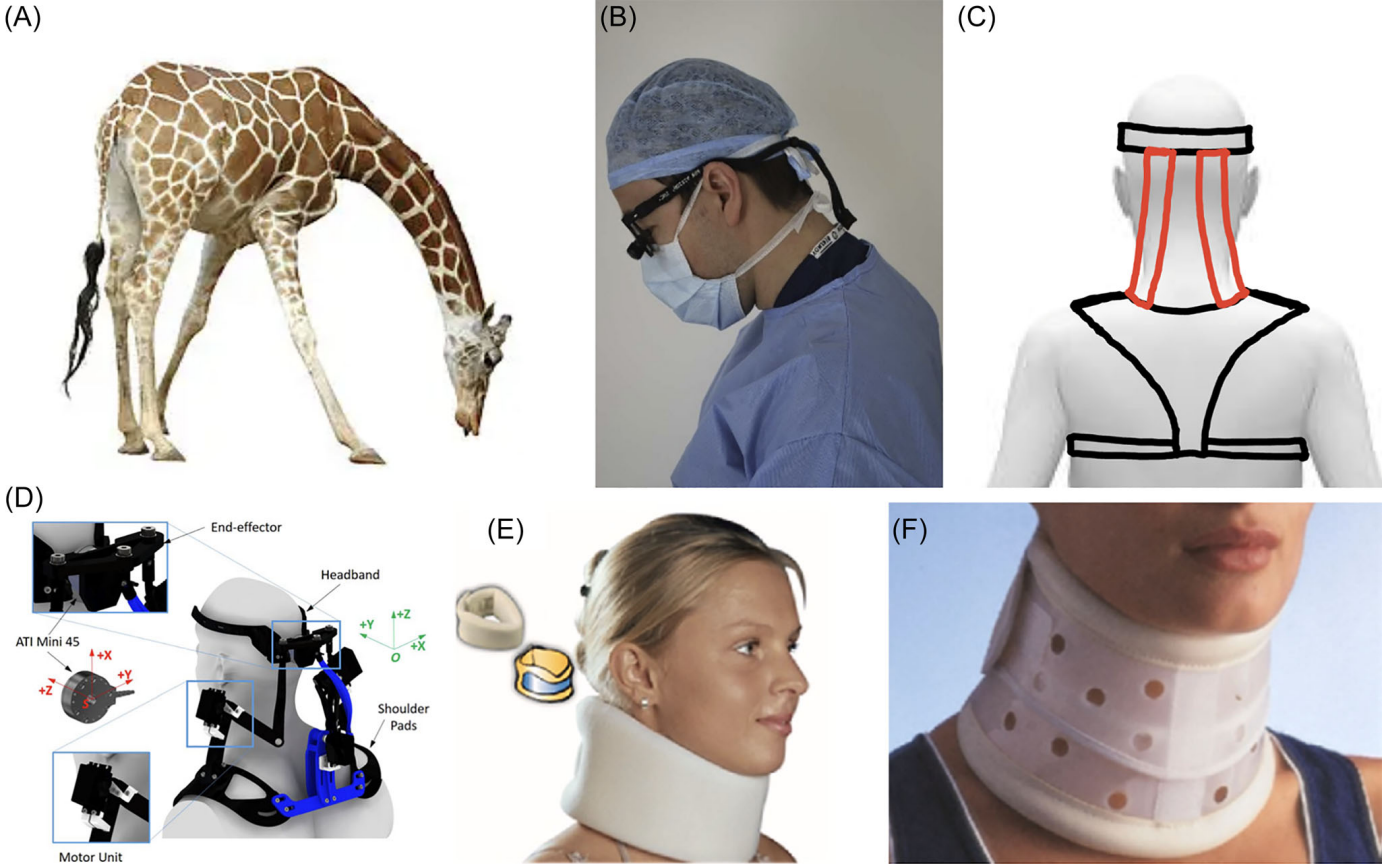
Dorsal–ventral flexion of the head and devices to resist this motion. (A) A giraffe's long neck creates large torques during ventral flexion. Muscle activity is reduced by the animal's strong nuchal ligament, which inspired this study. (B) A surgeons’ working posture. The neck is ventrally flexed to peer into a body cavity. Wearing loupes increases the weight of the head further. (C) Sketch of our passive neck brace in rear view, with the red elastic bands inspired by the giraffe nuchal ligament. (D) A motorized neck brace which reduces muscular activation when controlled by joystick. (E) A soft cervical collar that provides light stability and support. (F) A rigid cervical collar that prevents neck movement. (a) Panel A is reprinted from iStockPhoto.com (photographer: anankkml). Panel B is reprinted from Ref. 3. Panel D is reprinted from Ref. 24. Panel E is reprinted from Refs. 25, 26. Panel F is reprinted from Refs. 25, 27.

During surgery, surgeons peer downward into a body cavity, often for hours at a time. This posture involves dorsal‐ventral flexion of the neck (Figure 1) and in the long run is implicated in musculoskeletal disorders (MSDs).^1^ Symptoms of MSDs include recurrent pain, stiff joints, swelling, and dull aches.^2^ Among surgeons in the UK, 32% experienced cervical spine morbidity, and 28% required sick leave.^3^ Prevention of MSDs for surgeons mainly involves training for improved posture and strength. Loupes are recommended to be worn properly to reduce ventral flexion of the neck.^4^ Surgeons should pay attention to their neck, torso, and shoulder posture as well as perform conditioning to strengthen these body parts.^5^ Despite these risks, there are few wearable assistive devices (WADs) designed specifically for surgeons. The goal of this study is to develop a biologically inspired brace that supports the head in dorsal‐ventral flexion without restricting mobility in other directions.

Most braces are too restrictive to use by surgeons. Weightlifters and workers who pick up heavy objects wear a back brace, or lumbar support, for pain relief, immobilization, posture correction, and stabilization. For similar purposes, braces may be worn on the knee, elbow, and other joints. Figure 1D–F shows various WADs for the neck. A motorized neck brace supports head weight using external power, but also adds weight which may preclude use for long periods. While originally designed to keep patients from further injury, both rigid and soft cervical collars (Figure 1E,F) are lightweight enough to avoid weighing down the surgeon. However, the cervical collar is too mobility‐limiting and uncomfortable for surgeons to wear on the job. Specifically, it hinders lateral flexion and rotation, which is needed to exchange surgical tools, see computer monitors, and communicate with others at the operating table.

## MATERIALS AND METHODS

We built several prototypes, but our final design consists simply of a back‐brace posture corrector (Amazon, Selbite), a sports headband (Li‐ning), and two elastic bands (Michaels, Loops & Threads Non‐roll, elastic, 19.05 mm width). The elastic bands connect the headband to the back strap and are held in place by sewing, which was done by hand. The device was worn by three of the coauthors.

### Mathematical models

**FIGURE 2:**
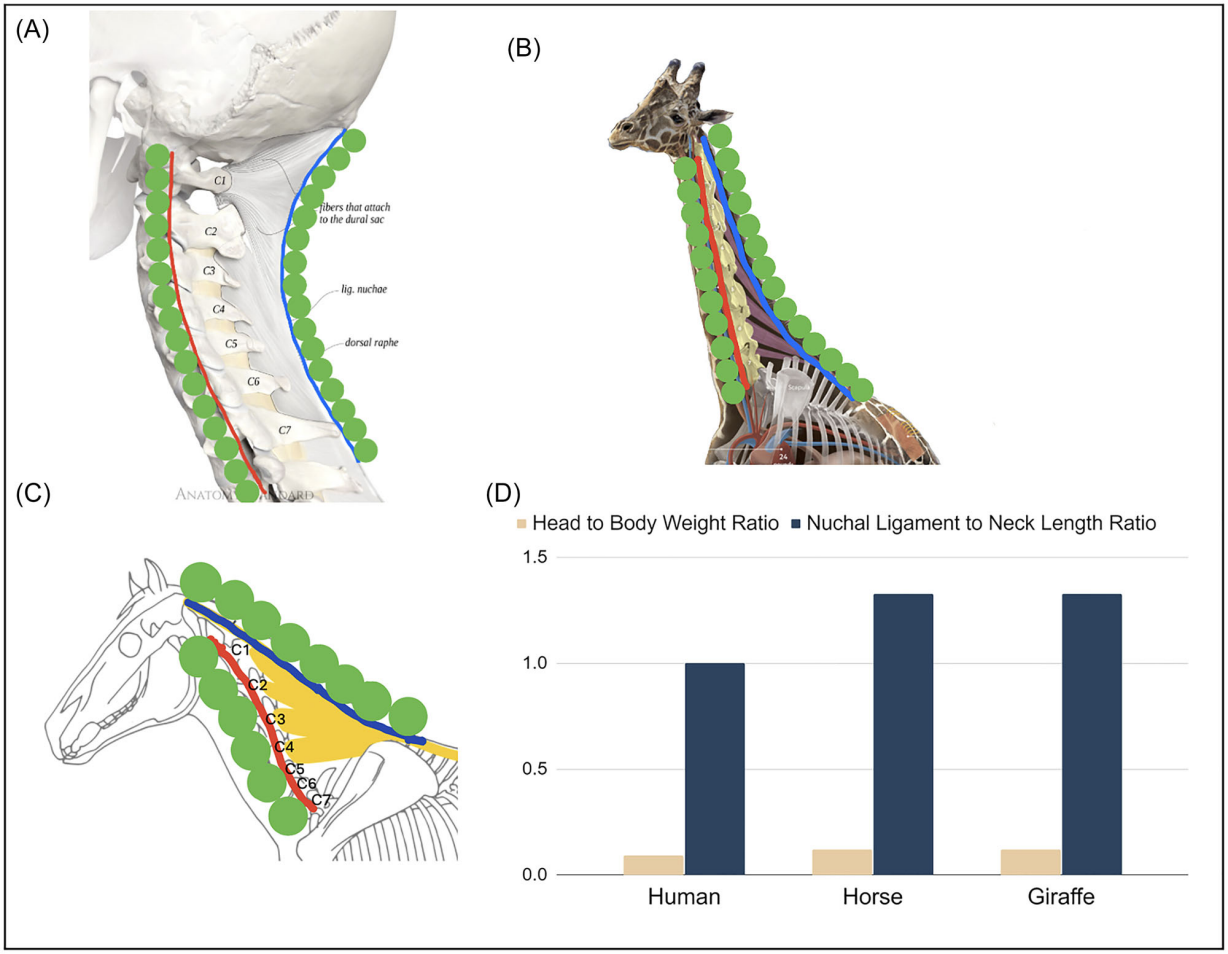
The nuchal ligament. (A) Human, (B) giraffe, and (C) horse. The red line shows the neck vertebrae and the blue line shows the funicular nuchal ligament. The green circles are shown to better visualize the arclength of neck vertebrae and nuchal ligament. C1–C7 denote the numbered cervical vertebrae. (D) Ratio of head weight to body weight (tan). Ratio of nuchal ligament length to cervical vertebrae length. Panel A is reprinted from Ref. 12, panel B is reprinted from Ref. 13, and panel C was drawn by Camille Saute (blog.equisense.com) based upon Refs. 9, 14.

In this section, we present a series of mathematical models to calculate the stretch of the funicular nuchal ligaments of a horse and a human during cervical flexion (Figure 2A,C). Inspired by studies of human head flexion during impact acceleration by the Navy,^6^ we model the horse neck using a one‐link pivot mechanism, and the human head with a two‐link pivot mechanism. We use these models to understand the best point of attachment of the brace.

### Model of the equine nuchal ligament

**FIGURE 3:**
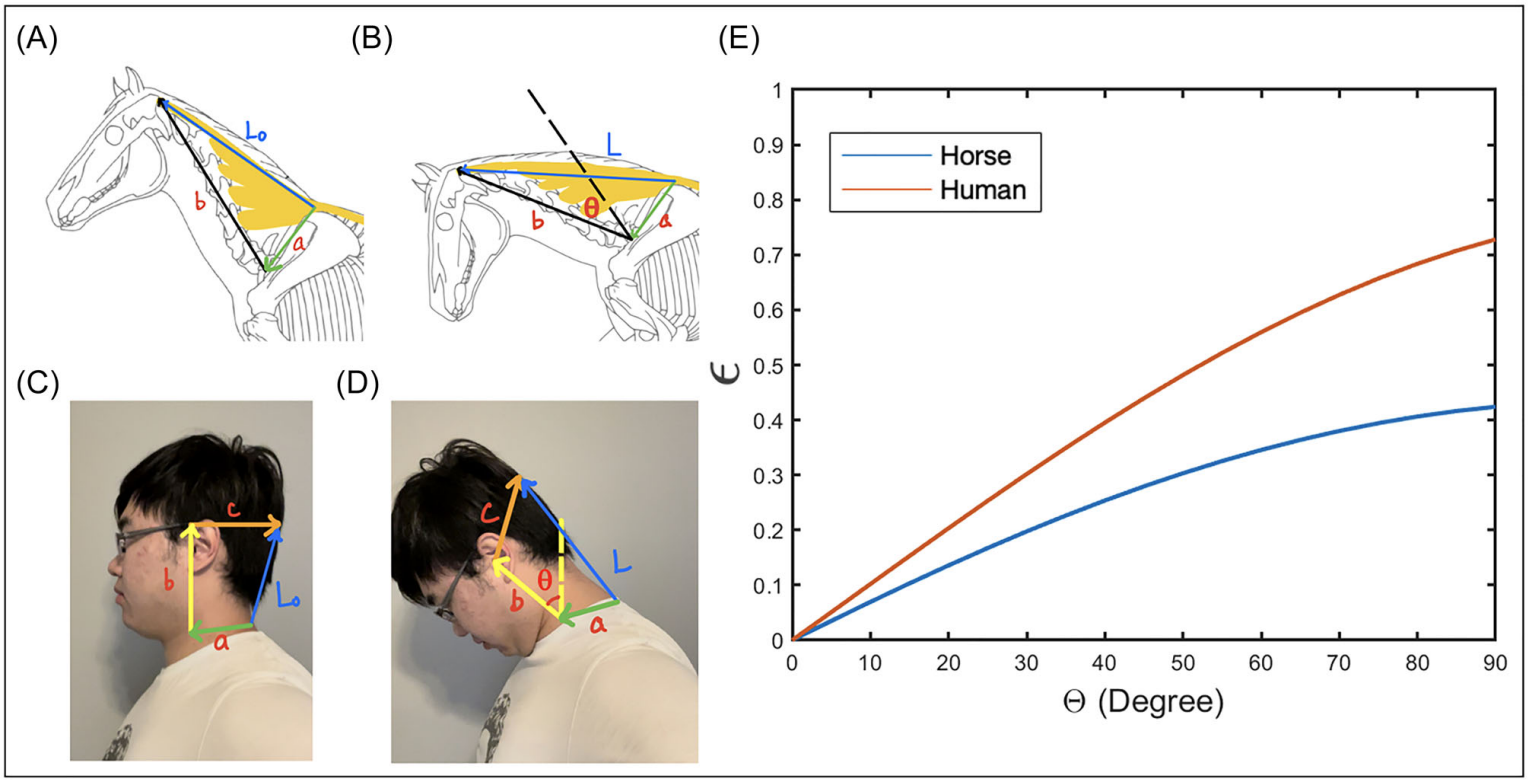
Schematic of neck flexion of (A, B) horse and (C, D) human. The vectors (**a**–**d**) are used to estimate the change in length of the nuchal ligament from *L*_0_ to *L*. (E) The relationship between nuchal ligament strain *ϵ* and flexion *θ* of the head, where blue is for the horse and red is for the human. Horse drawings (panels A and B) were modified from drawings by Camille Saute (blog.equisense.com) based upon Refs. 9, 14.

We consider the nuchal ligament of the horse, shown in blue and yellow in Figure 2C. The nuchal ligament is composed of a funicular, or cord‐like portion (blue), and a laminar portion (yellow). The funicular ligament runs parallel to the vertebrae, and the laminar portion has multiple connection points to the cervical bones. In our calculations of nuchal ligament strain, we focus only on the funicular portion, shown by the length of the blue line in Figure 3A,B. Thus, from here on, *nuchal ligament* will be used to refer to the funicular nuchal ligament.

The goal of this model is to determine the funicular ligament strain *ϵ* = (*L*(*θ*) − *L*_0_)*/L*_0_ as a function of the ventral flexion *θ* from its original neutral posture in Figure 3A. Here, *L*_0_ and *L* are the lengths of the funicular nuchal ligament in the neutral (Figure 3A) and flexed head posture (Figure 3B), respectively.

We assume flexion of the neck is due to joint‐like behavior at vertebrae C1 and C7 as shown in Figure 3. Indeed, C1 provides ventral‐dorsal flexion of 86 degrees, which is 32% of the range of motion of the equine cervical spine.^7^ The C1–C2 joint flexes 15 degrees, while the C7–T1 joint flexes 30 degrees. The cumulative flexion gives rise to the observed ventral flexion. Since the C1–C7 vertebrae flex less, for the purposes of our calculation, we treat the C1–C7 vertebrae as a straight line. We thus calculate the minimum strain of the nuchal ligament because accounting for curvature between C1 and C7 would add to the arc length and thus the strain.

The vector corresponding to the funicular nuchal ligament may be written as Equation (1):

(1) L=a+b,

where the vector **a** is fixed to the thorax, and thus maintains constant length and direction during cervical flexion. The start of **a** is at the vertebral processes in vertebrae T2–T3 where the nuchal ligament has major attachments. The end of **a** is the neck fulcrum at C7, which only has minor attachments of the nuchal ligament. Since our model's goal is only to estimate the stretch of the nuchal ligament, and not any forces, we can neglect any rotational moment due to attachments of the ligament at the endpoint of **a**. The vector **b** represents the neck vertebrae C1 through C7. It is fixed in length, but tilts counterclockwise by angle *θ* during flexion. Table 1 gives the measured magnitudes of **a**, **b**, and **c** of the male horse^8^ and the male human used in our calculation.

**TABLE 1:** Geometric variables for modeling nuchal ligament change in length.

	θ_*a*_ (°)	θ_*b*_ (°)	θ_*c*_ (°)	*a* (m)	*b* (m)	*c* (m)
Horse	225	120 + θ	N/A	0.25	0.62	N/A
Human	180	90 + θ	1.5 θ	0.09	0.27	0.15 m

*Note*: This table shows the geometric variables for modeling nuchal ligament change in length. The values for the horse are calculated using the proportions measured from Figure 4A and dimensionalized with the size of an average adult male horse from the Saklawi line.^8^ The length values for the human were measured on the lead author.

The origin of the coordinate system is set at the start of **a**. Based on the drawings of horse skeletons from Ref. 9, the angles of the vectors **a** and **b** may be written as Equations (2) and (3), respectively:

(2) $$\theta_{a}=225^{\circ },$$

(3) $$\theta_{b}=\theta +120^{\circ }.$$

Using **a** = *a*(cos*θ_a_* $\hat{x}$ + sin*θ_a_* $\hat{y}$), where *a* is the magnitude of the vector, we may use Equation (1) to write the length of the nuchal ligament as |**L**| = *L_x_* ^2^ + *L_y_* ^2^ where *L_x_* = *a* cos *θ_a_* + *b* cos *θ_b_*. Together, we may write the length of the nuchal ligament as Equation (4):

(4) $$L=\left|L\right|=\sqrt{{\left(a\;\sin\;\theta_{a}+b\;\sin\;\theta_{b}\right)}^{2}+{\left(a\;\cos\;\theta_{a}+b\;\cos\;\theta_{b}\right)}^{2}},$$

where we use Equation (3) to write it in terms of *θ*. The initial length of the nuchal ligament *L*_0_ is found using the condition *θ* = 0°.

### Model of human nuchal ligament

Here, we use similar methods to model the strain of the human funicular nuchal ligament (Figure 3C). Like the horse cervical vertebrae, much of the flexion range of motion is due to the first two cervical vertebrae, C1 and C2. The starting point of **a** indicates the funicular nuchal ligament attachment point at C7.

We initially tried to model the human nuchal ligament with a pair of line segments like the horse nuchal ligament, but the attempts led to errors in tracking the flexion. This is likely due to the shorter size of the human vertebrae and their greater curvature in flexion. Thus, we increased the complexity of our model by adding an additional segment to model flexion of the cervical spine. In this model, we keep the vectors **a** and **b** as defined in the previous section. We add a vector **c** that points from the center of mass of the skull to the ligament attachment point at the external occipital crest and the posterior tubercle of the atlas (C1). The human nuchal ligament vector may be written as the sum of the vectors in Equation (5).

(5) L=a+b+c

The human head's neutral position is vertical, as opposed to the horse's head in which neutral involves counterclockwise rotation of the vertebrae 45 degrees from the vertical. Moreover, in our observations of tracking the flexion of the human head, we found that the angle for **c** was dependent on the angle for **b**. By mapping the change in angle based on head flexion, we experimentally found the following relations in Equations ((6), (7), (8)):

(6) $$\theta_{a}=180^{\circ },$$

(7) $$\theta_{b}=90^{\circ }+\theta ,$$

(8) $$\theta_{c}=3\theta /2.$$

We filmed the first author's head in varying degrees of flexion and found that this model tracks the kinematics well. The length of the human nuchal ligament may be written as Equation (9):

(9) $$L=\left|L\right|=\sqrt{{\left(a\sin\theta_{a}+b\sin\theta_{b}+c\sin\theta_{c}\right)}^{2}+{\left(a\cos\theta_{a}+b\cos\theta_{b}+c\cos\theta_{c}\right)}^{2}}.$$

### Model of passive neck brace

**FIGURE 4:**
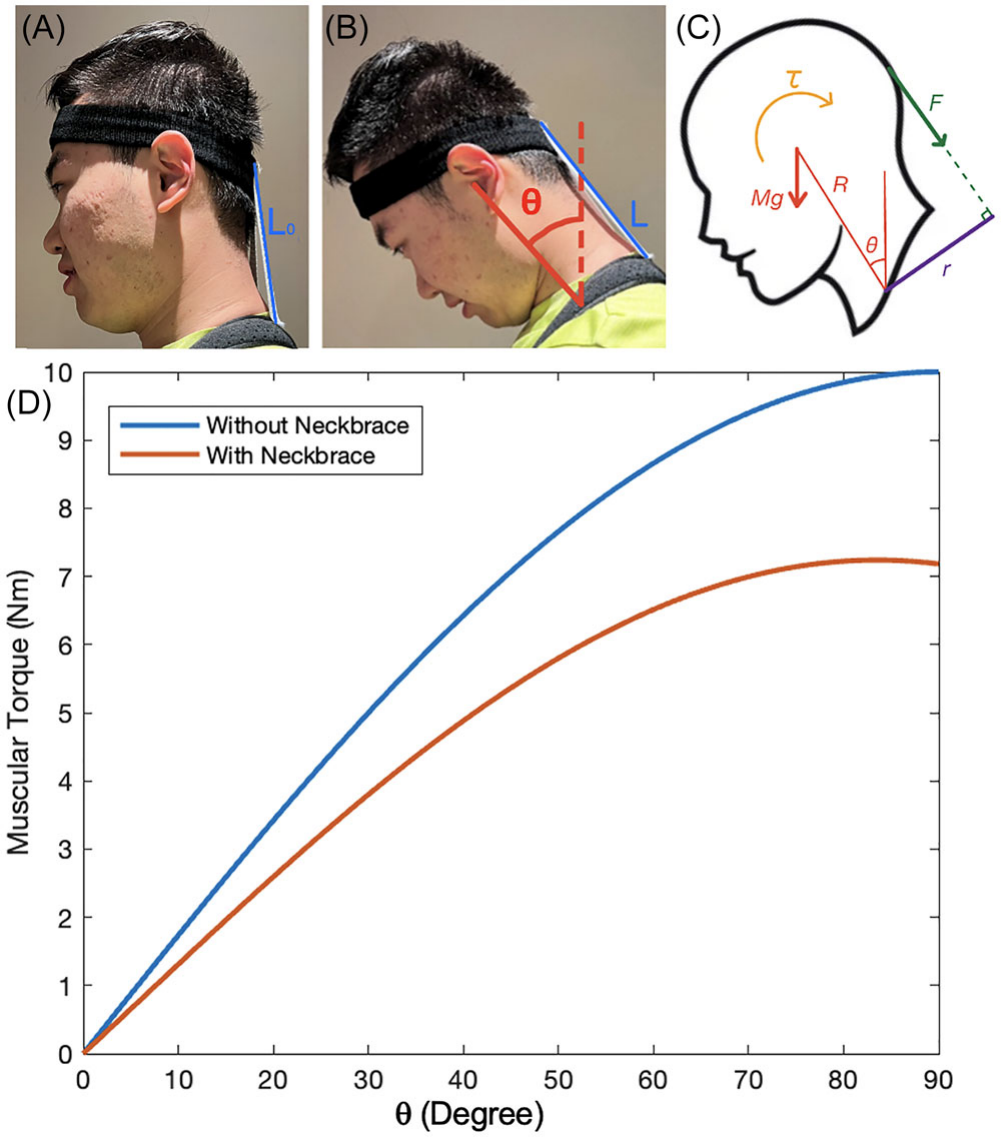
Side view of the lead author wearing the passive neck brace. (A) Head at neutral position. (B) Neck flexed ventrally *θ* degrees from the vertical. (C) Free body diagram of the head under ventral flexion. (D) The relationship between the calculated torque applied by muscles to keep head in equilibrium and flexion angle *θ*, where blue is without the passive neck brace and red is with passive neck brace.

Our next model estimates the reduction of muscular torque from wearing the passive neck brace. When the head is in a neutral position, shown in Figure 4A, the weight of the head is transmitted mostly vertically downward through the cervical vertebrae. Little muscular contraction is required to keep this posture. Ventral flexion of the neck requires the recruitment of muscles to balance torques. When the neck ventrally flexes by an angle *θ*, the head of mass *M* generates a gravitational torque *MgR*sin*θ* where *g* is the gravitational acceleration and *R* is the length of the cervical vertebrae. To counteract this torque, the neck muscles contract, applying a torque *τ* in the opposing direction. This isometric muscular contraction must be held as long as the neck is in flexion. The sustained isometric contraction is the primary reason for neck pain, so if we reduce the muscular contraction, we may be able to alleviate pain and injury.

Our device used elastic straps to apply a force *F* to resist rotation of the head, relieving some of the effort of the neck muscles. The headband and the shoulder strap keep the device at a distance *r* away from the fulcrum. This *r* is the mechanical advantage and reduces the force required by the elastic bands. The associated balance of the gravitational torque, neck brace torque, and muscular torque *τ* may be written as Equation (10):

(10) τ=MgRsinθ−Fr

The neck brace torque *Fr* may be calculated using our previous model for the nuchal ligament in Equation (9), which we used to estimate the elastic band length *L*(*θ*). To find the tension *F* in the band, we apply Hooke's Law, given in Equation (11):

(11) $$F=k\left(L-L_{0}\right),$$

where *L* is the current length of the band, *L*_0_ is its original length, and *k* is its spring constant. Figure 4D shows the muscular torque *τ* with (red) and without (blue) wearing a neck brace. Without wearing a neck brace, we set *F* = 0 in Equation (10). Without wearing a neck brace, we calculate the brace force using Equation (11) and *L* from Equation (9). When personalizing the head brace for the lead author, we decided to use two elastic bands. We measured *k* ≈ 160 N/m for two elastic bands in parallel. The bands were cut to length *L*_0_ = 15 cm. The distance between the headband and the shoulder brace is shown in Figure 4A. The length of the neck for the user in Figure 4 was *R* = 27 cm.

## RESULTS

Our device was inspired by ungulates such as horses and giraffes, which appear to easily flex their necks to graze on vegetation^10^ for 9–14 h per day. Many of these animals have long necks that extend away from the body. In this position, the torque from the head's weight is supported by a combination of the neck muscles and the nuchal ligament, a large elastic structure in the dorsal cervical region of the neck. Unlike most other ligaments, the nuchal ligament includes a good proportion of the protein elastin, allowing it to stretch to double its length without breaking.^11^

The blue bars in Figure 2D show the ratio of the nuchal ligament length to the neck length, measured from the hand‐drawn anatomical diagrams from Refs. 9, 12–14 and reprinted in Figure 2A–C. Horses have a nuchal ligament 25% longer than their cervical neck vertebrae. The ligament attaches at the withers, the highest point on the horse's back, and then continues caudally as the supraspinous ligament. The giraffe nuchal ligament is also about 25% longer than its neck vertebrae. The giraffe's back has a small hump: it is indicative of the larger spinal processes to increase the area of contact with the laminar nuchal ligament, necessary to support the giraffe's neck, which can be 6 feet long and weigh up to 600 pounds.^15^

In addition to the ungulates, dogs also have a nuchal ligament and are the only carnivore to do so. The nuchal ligament allows them to lift their head high while running long distances as well as to keep their nose to the ground while following scent trails.^16^ The canine nuchal ligament differs anatomically from that of ungulates. In ungulates, the ligament attaches to the back of the skull, but in the dog, it attaches to the back of the axis, the second cervical vertebra. In general, strong nuchal ligaments are more the exception than the rule. In most other mammals, the nuchal ligament is absent or very small, including in the great apes. Humans have a nuchal ligament that matches their neck vertebrae length. The human nuchal ligament is thus relatively shorter than that of horses and giraffes. Humans evolved an upright posture, where the head does not generate much gravitational torque, as the weight of the head is nearly supported from below by the cervical vertebrae, as shown in Figure 2A. Consequently, humans have shorter and weaker nuchal ligaments that lead to MSDs when the neck is flexed for long times, as for surgeons at work.

The gravitational torque from the head is a product of the head weight and the horizontal displacement of the head from the body. One would think that ungulates have a relatively heavier head for their body size. The body weights of an adult human, horse, and giraffe are approximately 80, 500, and 1200 kg, respectively.^17^, ^18^ The head weights are all roughly 0.1–0.2 proportion of the body weight.^19^ Thus, the length of the neck, which provides a longer lever arm, is the primary reason why ungulates evolved a stronger nuchal ligament.

To demonstrate that the horse's nuchal ligament attachments evolved to reduce strain, we compared the strain due to flexion of the horse and the human nuchal ligament. The lines in Figure 3 show our calculations of the strain *ϵ* = (*L* − *L*_0_)*/L*_0_ of a horse (red) and human (blue) nuchal ligament. Inputs to the model are the neck and shoulder anatomy, such as the neck length and distance from the neck fulcrum to the nuchal ligament attachment point. When bending the head 40 degrees, the horse has a strain of *ϵ* = 0.25 and the human of 0.4. We were unable to calculate muscular torque without measurement of the nuchal ligament material properties. Nevertheless, we surmise the lower strain of the horse nuchal ligament may help reduce neck pain for the horse.

Inspired by the mechanical advantage of the horse nuchal ligament, we designed a passive neck brace, a wearable device to augment the human nuchal ligament. Figure 4 shows how the neck brace resists the gravitational torque by the head. Compared to the neck brace, the human's nuchal ligament is located closer to the spine, resulting in a reduced *r* value shown in Figure 4A. This *r* value is the lever arm over which the brace applies the torque. A larger *r* for the neck brace means a greater mechanical advantage.

Our passive neck brace consists of a headband linked to a posture corrector back brace by elastic straps. These attachment points lay outside the body and so increase the value of *r* beyond the anatomical limits of the human neck. Increasing the mechanical advantage *r* is a common principle among ungulates, as shown by the large *r* associated with the funicular nuchal ligament, given by the distance between posterior endpoints of the red and blue lines, shown in Figure 2B,C. The precise calculation of mechanical advantage for the horse and giraffe is complicated by the laminar portion of the nuchal ligament.

Flexing the neck ventrally increases gravitational torque. Surgeons might flex their necks up to *θ* = 60 degrees to peer into a body cavity. The blue line in Figure 4D shows the muscular torque without wearing the head brace. Increasing the angle from 10 to 60 degrees increases the required muscular torque by a factor of eight. In response, the neck muscles need to contract with eight times as much force to keep the head in equilibrium.

The red line in Figure 4D shows the muscular torque when the neck brace is worn. The more torque provided by the neck brace, the less torque needs to be provided by the muscles to hold the neck in position. When the head is flexed 40 degrees, the elastic bands stretched by 20% (strain *ϵ* of 0.198), and the neck muscles need only resist 28.3% of the gravitational torque, as shown by the difference between the red and blue lines.

We originally thought that the head brace should resist 100% of the gravitational torque, reducing the neck muscle contraction to zero. We wore braces of various stiffness of the elastic bands. We found the stiffer brace to be uncomfortable because it required perceptible effort to flex the neck. Thus, when tuned for comfort, the red line corresponds to the greatest brace stiffness that could be accommodated.

## DISCUSSION

One drawback of the device is that the headband begins to slip off the head, as shown in Figure 4. This makes sense as the headband was not designed to support a shear force applied to one side of the band. There may be ways to design the band to resist sliding. In the meantime, future users might add tightening mechanisms to keep the headband on and adjust it for other users. We also note that the sliding also helped increase the comfort of the device, at the cost of reducing its stability. To allow the elastic strap length *L*_0_ to be adjusted for different users, Velcro or buttons may be used. A stiffer brace could help train correct posture, similar to a cervical collar. Wearing the brace may induce stress on the skin, which may be measured using soft sensors.^20^

Our design of the neck brace focused on simplicity, but future designs should consider the surgeon's other constraints. First, surgeons have limited dress time before they operate, requiring the device to be quickly and easily worn. Moreover, the sterile technique prevents surgeons from touching any nonsterile surfaces once they are scrubbed in.^21^ This means that if at any point the surgeon feels uncomfortable with this device, they will need other people to adjust or remove the device for them. Future versions might have pneumatics or some other ways to adjust the device stiffness with a single touch on the chest or with voice control. Such a design would increase the device's weight through motor, batteries, and other complexity. Increasing the weight of the device would have to be done carefully because this extra weight would be borne by the surgeons, which might further cause poor posture.

We envision this device to help in the short term with neck pain and in the long term to prevent MSDs. MSDs are called cumulative trauma disorders because they are gradual onset injuries that occur from repeated imperceptible microtraumas to a particular body part.^22^ They may take weeks, months, or years to develop, and are often ignored because of the slow onset of symptoms. We envision that the passive neck brace can be used as a supplement to daily surgical practice. Short‐term use of the brace may help, but neck braces are only recommended for long‐term use if protecting the neck from further damage outweighs the consequences of use. Long‐term use of a brace may cause muscle weakness.^23^ Thus, physicians recommend limiting brace use and prescribing physical therapy exercises as soon as possible. One potential application of the passive neck brace would be to help surgeons recovering from MSD ease back into work.

The device was worn by three of the coauthors, one of whom is an interventional radiologist. The radiologist wore the brace and moved his head around a few times and held his head down to feel the tension from the brace. The radiologist stated “this device provides forces in the opposite direction of head deflection and thus is functional considering the design objective. The device does not limit the head movement, which is important for the user experience.” In addition, the first author wore the device for 10 min and did not find any discomfort besides the compression provided by the headband and posture protector. Future clinical trials are necessary with a range of individuals, including those predisposed for MSDs, such as people with obesity. Future studies will have to be done to see if the following symptoms occur: rashes, vomiting sensation, dizziness, and obstruction of blood circulation.

Hygiene studies would have to be conducted to wear the device in the operating room. There may be other conditions that result from wearing the passive brace for hours at a time. The passive brace is composed of the posture corrector, the headband, and the elastic bands. The posture corrector is suggested to be worn by the manufacturer for 6 h a day.

This device is still in technology readiness level (TRL) of 2–3, where TRL is a metric to assess the maturity of a technology. For any device like a brace for a body part, there will have to be an FDA regulation pathway, cooperation with companies on mass manufacturing, and potential clinical studies if the device is found to provide medical treatment.

## CONCLUSION

Our goal was to design a simple lightweight neck brace that passively reduced the muscular effort to hold the head in a ventrally flexed position. Using the horse's strong nuchal ligament as inspiration, we built a passive neck brace for under $40. User interviews and focus groups are needed to improve the device. We hope this work increases the visibility of bioinspired design and surgical ergonomics.

## AUTHOR CONTRIBUTIONS

Z.Y. conducted all the literature reviews, prototype construction and verification, and cowrote this paper. T.S.S. and M.S. provided clinical needs and advice during the early stage of the research. J.H.S. provided feedback and suggestions on the prototype. D.L.H. supervised the project and cowrote the paper.

## COMPETING INTERESTS

The authors declare that they have no competing interests.

### PEER REVIEW

The peer review history for this article is available at https://publons.com/publon/10.1111/nyas.15308.
